# NSAIDs Ibuprofen, Indometacin, and Diclofenac do not interact with Farnesoid X Receptor

**DOI:** 10.1038/srep14782

**Published:** 2015-10-01

**Authors:** Jurema Schmidt, Franca-Maria Klingler, Ewgenji Proschak, Dieter Steinhilber, Manfred Schubert-Zsilavecz, Daniel Merk

**Affiliations:** 1 Institute of Pharmaceutical Chemistry, Goethe-University Frankfurt, Max-von-Laue-Str. 9, 60438 Frankfurt, Germany

## Abstract

The nuclear farnesoid X receptor (FXR) is a ligand activated transcription factor and acts as cellular sensor for bile acids. In this role, FXR is a highly important liver protector and FXR inhibition by antagonists or knockout has shown several deleterious effects. A recent report characterized non-steroidal anti-rheumatic drugs (NSAIDs) such as ibuprofen or diclofenac as FXR antagonists and linked hepatotoxic effects of these drugs with antagonistic activity on FXR. Since this would guide a way to develop safer anti-inflammatory agents by sparing FXR, we intended to further characterize the reported antagonistic activity and intensively investigated ibuprofen, indometacin and diclofenac. However, we conclude that these agents do not interact with FXR and that the reported reduced FXR signaling induced by CDCA in presence of NSAIDs is merely a consequence than a cause of hepatotoxicity.

Nuclear farnesoid X receptor (FXR) is a crucial ligand-activated transcription factor acting as bile acid sensor^1^,^2^,^3^. As such, FXR regulates the homeostasis of bile acids and is a highly important liver protector^4^. Upon FXR activation by e.g. high levels of toxic bile acids, bile acid synthesis is blocked and bile acid metabolism and excretion are enhanced^4^,^5^. Thereby FXR shields liver cells against the accumulation of bile acids and their toxic effects. FXR is furthermore involved in several other metabolic regulatory systems including lipid and glucose homeostasis and seems to have anti-inflammatory activity as well^5^.

Physiologically, FXR is activated by bile acids of which chenodeoxycholic acid (CDCA) is the most potent FXR agonist with an EC_50_ value of about 8 μM^3^. Intensive research within the last decade has also discovered several synthetic FXR agonists and antagonists^6^,^7^. However, at present only FXR agonism seems to have a therapeutic potential while FXR antagonism is rather associated with undesired effects^8^. FXR activation is currently evaluated as therapeutic concept for the treatment of severe liver disorders such as non-alcoholic steatohepatitis (NASH) and primary biliary cirrhosis (PBC) with obeticholic acid (OCA) as experimental FXR agonist in advanced clinical development^9^,^10^,^11^.

Recently, Lu *et al*.^12^ have reported NSAIDs to have antagonistic activity on FXR and they state that this antagonism contributes to liver damage caused by chronic intake of NSAIDs. This data intrigued us for two particular reasons. First, would FXR antagonism actually contribute to toxicity and side effects of NSAIDs, this knowledge would suggest an opportunity to develop safer anti-inflammatory agents by designing cyclooxygenase inhibitors that spare FXR. Second, FXR antagonists are rare but required as pharmacological tools for further investigation of the physiological and pathophysiological roles of FXR. The small and highly drug-like NSAIDs would be a very valuable starting point for the development of potent FXR antagonists.

However, we were confounded by the low maximum FXR activation in the test systems reported by Lu *et al*. and the fact that chemically quite diverse agents such as the NSAIDs diclofenac and indometacin show comparable activity on FXR. Therefore, we intended to reproduce and further characterize the reported data in our test systems and selected the NSAIDs ibuprofen, indometacin, and diclofenac to cover a high structural variety amongst the NSAIDs (Fig. 1). We applied these three NSAIDs to several test systems including a full-length reporter gene assay, a hybrid reporter gene assay, quantitative real-time PCR, and thermal shift experiments. To our surprise, none of the examined NSAIDs exhibited any activity or binding on FXR.

## Results

### Hybrid reporter gene assay

First, we characterized ibuprofen, indometacin, and diclofenac in a hybrid FXR reporter gene assay^13^ in COS-7 cells for agonistic and antagonistic activity. The assay is based on a fusion receptor of the FXR ligand binding domain and a Gal4 DNA binding domain from yeast. As reporter gene the assay contains a firefly luciferase under the control of a Gal4 response element which is only transcribed upon activation of the hybrid receptor. A renilla luciferase with a constitutively active SV40 promoter serves as control for transfection efficiency and toxicity. The assay was validated with FXR agonists CDCA (EC_50_ = 14.5 ± 0.8 μM), GW4064 (EC_50_ = 0.22 ± 0.04 μM) and OCA (EC_50_ = 0.54 ± 0.05 μM) which showed values in good agreement with the literature^9^,^14^,^15^,^16^. As antagonistic reference the commonly used selective bile acid receptor modulator (SBARM) guggulsterone^8^ was employed which in this test system had an IC_50_ value of 13.6 ± 2.8 μM. In order to determine antagonistic activity, test compounds were co-incubated with GW4064 (3 μM), OCA (3 μM) or CDCA (20 μM).

**Figure 2:**
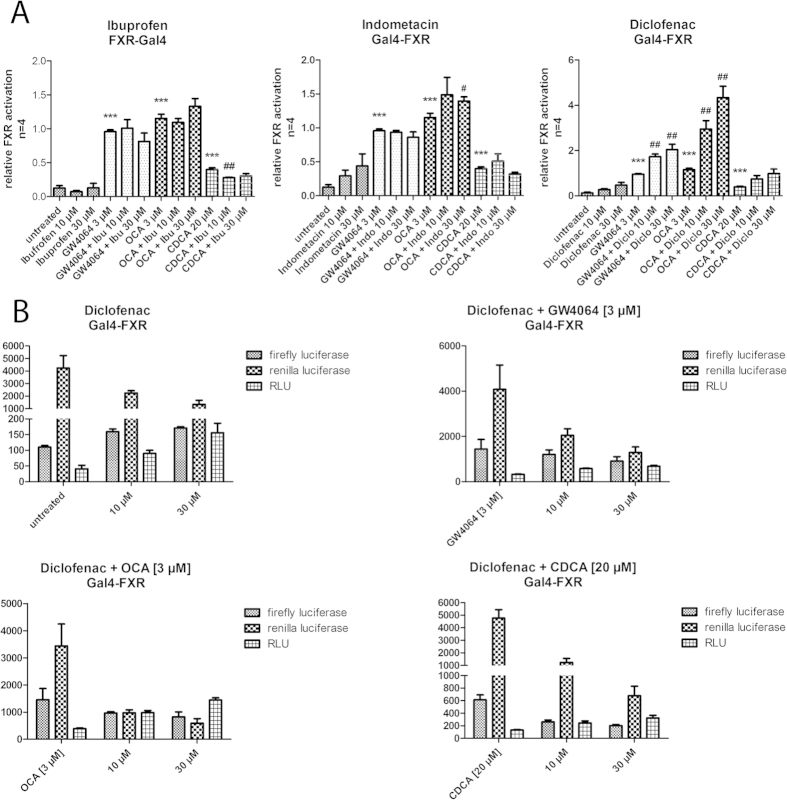
FXR-Gal4 hybrid reporter gene assay (data represents mean ± SEM; n = 4). (**A**) None of the NSAIDs ibuprofen, diclofenac and indometacin showed any agonistic or competitive antagonistic activity in the hybrid reporter gene assay. Compounds were assessed at 10 or 30 μM alone and in competition with GW4064 (3 μM), OCA (3 μM) and CDCA (20 μM). (**B**) The increased relative activity of diclofenac (suggestive of FXR agonism) does not result from increased reporter (firefly luciferase) activity but from a toxicity driven decrease in the activity of the control gene (renilla luciferase). The effect does therefore not represent an (agonistic) activity on FXR. (*p < 0.05; **p < 0.01; ***p < 0.001).

In the Gal4-FXR hybrid assay, none of the tested NSAIDs showed any agonistic or antagonistic activity at concentrations of 10 μM and 30 μM which is far above the reported IC_50_ values (Fig. 2). Notably, the concentration of CDCA was quite low with 20 μM and therefore it could be expected that the NSAIDs would have even higher potency than in the reported data where the NSAIDs had to compete with 50 μM CDCA. For diclofenac, the assay data on first glance would indicate an additive agonistic activity that enhances the potency of all FXR agonists used. However, this effect was due to a cytotoxic effect that only negatively affects the control gene without enhancing the expression of the reporter gene (Fig. 2).

### Full-length reporter gene assay

Next, we assumed that eventually the entire FXR receptor protein might be required for the reported activity of the NSAIDs and therefore, we continued the evaluation in a full-length FXR reporter gene assay under the same competitive conditions with GW4064, OCA and CDCA. The assay was conducted in HeLa cells and is based on the complete FXR receptor (CMV promoter) and its hetero-dimer partner retinoid X receptor (RXRα, CMV promoter). As reporter gene, a firefly luciferase under the control of a bile salt export protein (BSEP) promotor was used and a renilla luciferase (SV40 promoter) served as transfection and toxicity control^17^. The assay was also validated with FXR agonists CDCA (EC_50_ = 18 ± 1 μM), GW4064 (EC_50_ = 0.51 ± 0.16 μM) and OCA (EC_50_ = 0.16 ± 0.02 μM), which yielded values in good agreement with the literature^9^,^14^,^15^,^16^.

**Figure 3:**
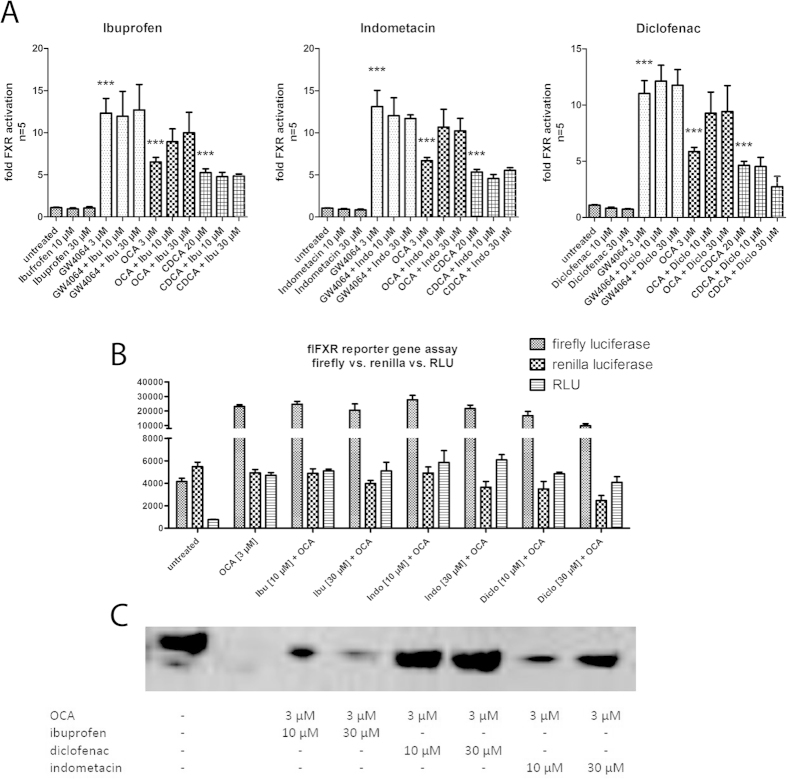
Full-length FXR reporter gene assay (data represents mean ± SEM; n = 5). (**A**) None of the NSAIDs ibuprofen, diclofenac, and indometacin showed any agonistic or competitive antagonistic activity in the full-length FXR reporter gene assay. Compounds were assessed at 10 or 30 μM alone and in competition with GW4064 (3 μM), OCA (3 μM) and CDCA (20 μM). (**B**) The increased relative activity of NSAIDs when combined with OCA (3 μM) (suggestive of FXR agonism) does not result from increased reporter (firefly luciferase) activity but from a toxicity driven decrease in the activity of the control gene (renilla luciferase). The effect does therefore not represent an (agonistic) activity on FXR. (**C**) Western blotting on β-actin confirms the toxic activity of OCA and NSAIDs in combination (normalized on cell number). (*p < 0.05; **p < 0.01; ***p < 0.001).

In the full-length FXR reporter gene assay again, none of the tested NSAIDs showed any agonistic or antagonistic activity (Fig. 3A). For the competition with OCA, we observed a slight increase in relative light units which was due to a toxicity driven decrease in activity of the control gene (renilla luciferase) but not to an effect on the firefly luciferase as reporter gene (Fig. 3B). This assumed toxic effect was also confirmed when we used the assay cells for western blotting on the house keeping gene β-actin which is quite sensitive for toxicity (Fig. 3C). Hence, NSAIDs seemed to have an increased toxicity in HeLa cells when combined with obeticholic acid (Fig. 3).

### Toxicity

In the full-length FXR reporter gene assay and in the Gal4-FXR hybrid reporter gene assay we observed considerable toxicity for the NSAIDs. Especially the combination of NSAIDs with competitor significantly affected the viability of the assay cells in several cases. Therefore, we analyzed the toxicity of ibuprofen, indometacin and diclofenac under various conditions using the WST-1 assay.

**Figure 4:**
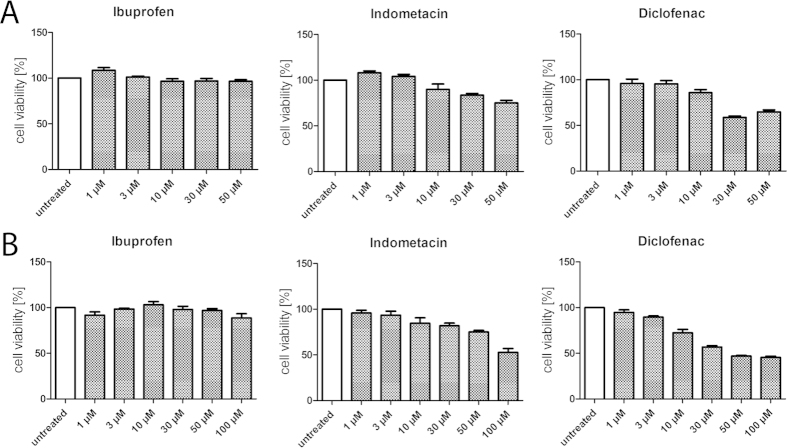
Toxicity of NSAIDs in HepG2 cells (**A**) and HEK293T (**B**) cells (data represents mean ± SEM; n = 4). In HepG2 cells (**A**) indometacin and diclofenac showed slight toxicity starting from a concentration of 10 μM while ibuprofen was non-toxic up to 50 μM. In HEK293T cells (**B**) the toxicity of indometacin and especially diclofenac is enhanced.

In HepG2 cells, ibuprofen showed no toxicity up to 50 μM whilst both indometacin and diclofenac generated anti-proliferative activity at concentrations of 10 μM and above. In HEK293T cells which Lu *et al*. have used for their reporter gene assay the NSAIDs indometacin and diclofenac exhibited even higher toxicity (Fig. 4).

**Figure 5:**
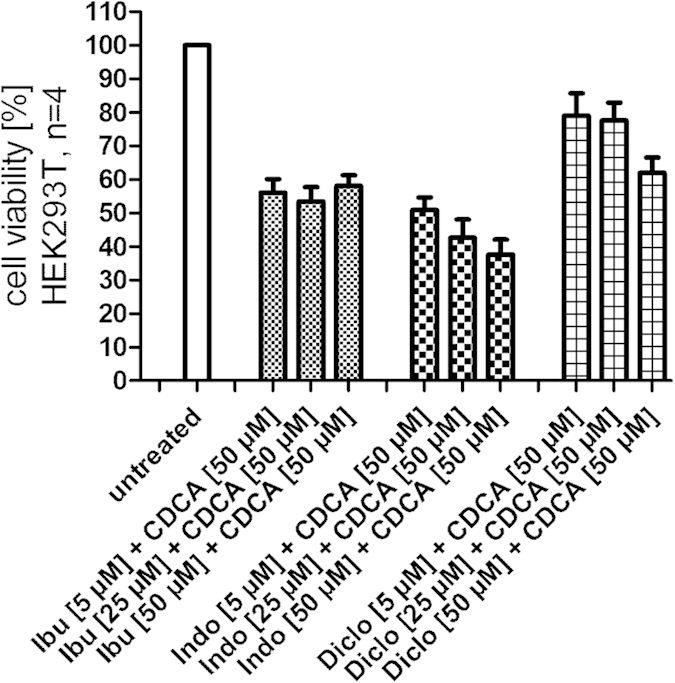
Toxicity of NSAIDs in combination with CDCA in HEK293T cells (data represents mean ± SEM; n = 4). Ibuprofen, diclofenac and indometacin exhibit considerable anti-proliferative effects on HEK293T cells when combined with CDCA (50 μM). This activity may result in reduced reporter gene expression in test systems but must not be interpreted as antagonism.

When the NSAIDs are combined with CDCA which is necessary for evaluation of competitive antagonism, the toxicity is strongly enhanced. In consequence, cells that are e.g. used for a reporter gene assay and treated with a combination of 50 μM CDCA and a NSAID are significantly restricted in their viability. This in turn affects the results of the assay. As a result of increased toxicity the reporter gene assay indicates lower FXR activity which must not be interpreted as antagonism since it is not a result of an activity on target. To circumvent this problem, lower concentrations of CDCA as competitor in a reporter gene assay should be used for less robust cell lines. When we evaluated the cell viability of HEK293T cells with the WST-1 assay after incubation with NSAIDs (5 μM, 25 μM and 50 μM) in combination with CDCA (50 μM) we received almost exactly the same picture as Lu *et al*. reported for their reporter gene assay in HEK293T cells. This might eventually explain the reported data since we treated the cells almost equally as Lu *et al*. except that we did not transfect them for a reporter gene assay (Fig. 5).

**Figure 6:**
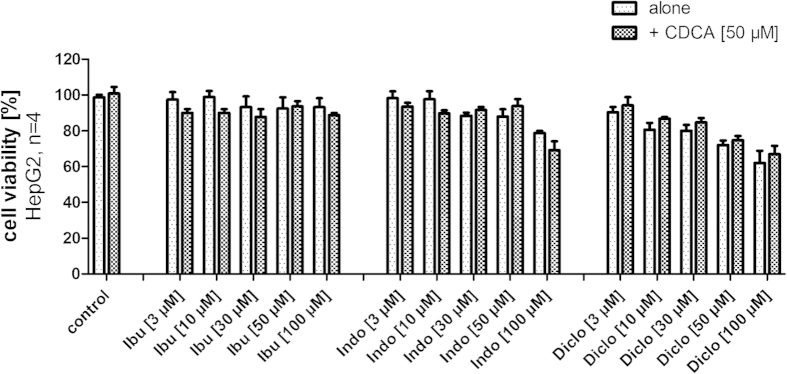
Toxicity of NSAIDs alone or in combination with CDCA in HepG2 cells after a short incubation period of 6 hours (data represents mean ± SEM; n = 4). With shorter incubation, the toxicity of NSAIDs is reduced and for all NSAIDs concentrations of 30 μM and 50 μM seem suitable for experiments with 6 hours incubation time.

We assumed that shorter incubation periods might reduce toxic effects in test systems which would help to generate more robust data. Therefore, we also determined cell viability of HepG2 cells after 6 hours of incubation with NSAIDs (3 μM–100 μM) alone or in combination with CDCA (50 μM, Fig. 6). For ibuprofen and indometacin, the shorter incubation period could nearly completely prevent toxicity. Ibuprofen showed no significant toxic effects up to 100 μM while indometacin did not affect cell viability up to 50 μM. Also diclofenac showed less toxic effects but the effect was less pronounced. Still, with 85 ± 2% cell viability compared to untreated control for the combination of 30 μM diclofenac and 50 μM CDCA and 75 ± 2% for 50 μM diclofenac and 50 μM CDCA both concentrations seemed suitable and interpretable after 6 hours of incubation.

Summing up, indometacin and especially diclofenac are considerably toxic at concentrations of 30 μM or higher. This toxicity is strongly enhanced when the compounds are co-incubated with CDCA. Hence, additive toxic effects may explain variations in experimental data which must not be misinterpreted as antagonism. Shorter incubation periods can reduce the toxic effects which may help to generate more robust data.

### FXR target gene quantification (qRT PCR)

**Figure 7:**
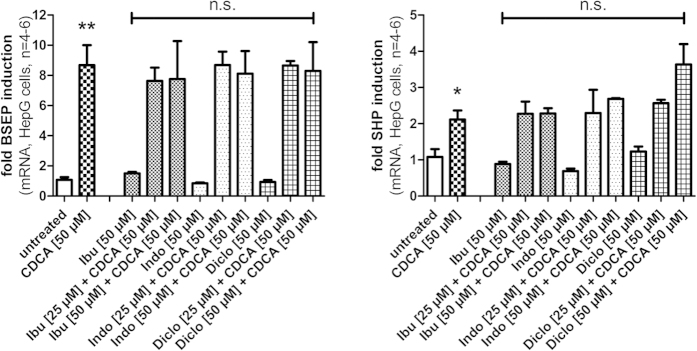
FXR target gene quantification by qRT-PCR (data represents mean ± SEM; n = 4–6). Ibuprofen, diclofenac and indometacin (all 50 μM) did not affect the expression of the direct FXR target genes bile salt export protein (BSEP) and small hetero-dimer partner (SHP) in HepG2 cells after 6 h incubation. Furthermore, none of the NSAIDs (25 μM and 50 μM) antagonized the effect of CDCA (50 μM) on the expression of BSEP or SHP. (*p < 0.05; **p < 0.01; ***p < 0.001; n.s.—not significant).

Quantification of the expression of FXR target genes in non-transfected cells offers the least artificial way to characterize the activity of a FXR ligand *in vitro*. Since FXR activity is especially important in liver, hepatocytes are suitable to evaluate effects of test compounds on FXR target genes. We therefore investigated the activity of NSAIDs ibuprofen, diclofenac, and indometacin in HepG2 cells. As investigated genes we selected the direct FXR target genes bile salt export protein (BSEP) and small hetero-dimer partner (SHP) which allows a specific conclusion whether a FXR-dependent effect is present. To reduce toxic effects we incubated the cells with the NSAIDs and CDCA for 6 hours which is certainly enough to see effects on direct FXR target genes such as BSEP and SHP. Previous toxicity determination in HepG2 cells after 6 hours incubation with NSAIDs and CDCA (Fig. 6) confirmed this assumption since no strong toxicity occurred. To further exclude toxic effects, we selected two different concentrations of NSAIDs for the evaluation of FXR target gene expression and tested all NSAIDs in 25 μM and 50 μM concentration in competition with CDCA (50 μM). According to our toxicity determination, the combination of ibuprofen, indometacin or diclofenac in 25 μM concentration and CDCA (50 μM) did not affect cell viability after 6 hours incubation. We then quantified the mRNA of FXR target genes BSEP and SHP by qRT-PCR. Incubation of HepG2 cells with the NSAIDs (25 μM, 50 μM) alone or in competition with the endogenous FXR agonist CDCA (50 μM) revealed no significant activity and confirmed the results from the reporter gene assays. None of the NSAIDs in 50 μM concentration had any significant agonistic or antagonistic effect on BSEP or SHP expression alone and none of the NSAIDs significantly reduced the effect of CDCA on the expression of BSEP or SHP (Fig. 7).

### Thermal shift

Although all *in vitro* data contradicted an interaction of the NSAIDs ibuprofen, diclofenac and indometacin with FXR we also intended to determine binding of NSAIDs to the FXR ligand binding domain (LBD). Lu *et al*.^12^ have postulated by in silico investigations that NSAIDs directly interact with the ligand binding site of FXR. Moreover, investigation of a direct interaction between potential ligand and target protein excludes any artifacts caused by the cellular background in reporter gene assays or qRT-PCR experiments.

For a nuclear receptor such as FXR, activation by a ligand is facilitated by interaction of the ligand with the ligand-dependent activation function 2 (AF-2) which is located in helix 12 of the LBD. The interaction with AF-2 causes a recruitment and stabilization of helix 12 to the rest of the LBD. In consequence, a new surface is generated on the LBD where co-activator peptides can be bound. An antagonist on a nuclear receptor in contrast prevents the stabilization of helix 12. We assumed that an increased stability of the FXR-LBD that is facilitated by direct binding of a ligand could be determined in thermal shift experiments.

The thermal shift method is widely used to assess ligand binding^18^,^19^. A thermal shift assay usually measures the melting temperature of a protein with or without a ligand by monitoring a fluorescent dye. In an aqueous solution of folded protein the dye signal is low due to quenching of the dye fluorescence in water, but with increasing temperature the protein unfolds and exposes its hydrophobic core regions. The fluorescent dye is then bound to the hydrophobic surfaces and fluorescence increases^18^,^19^.

**Figure 8:**
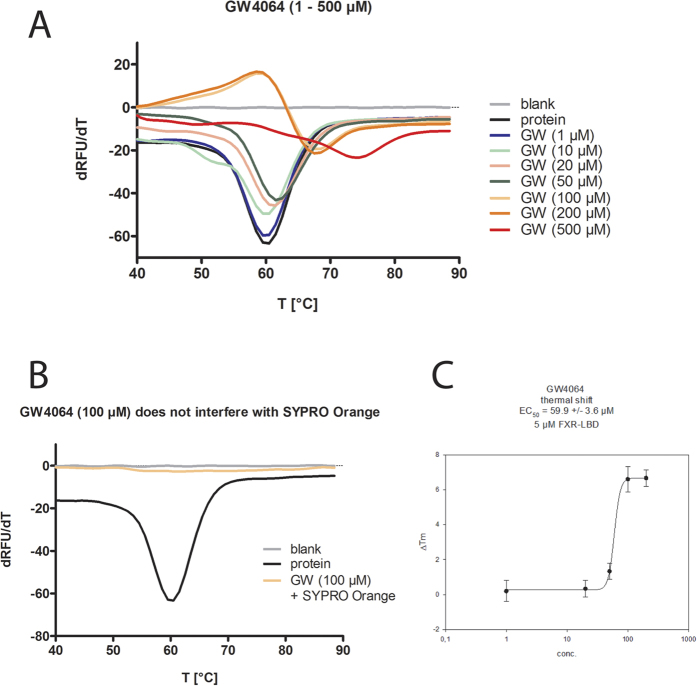
Validation for thermal shift experiments. (**A**) FXR agonist GW4064 robustly stabilizes the FXR-LBD at concentrations ≥100 μM (ratio to protein ≥20:1) and produces a significant thermal shift. (**B**) GW4064 showed no interaction with the dye SYPRO Orange. (**C**) GW4064 produced a robust thermal shift of about 6 °C and with our conditions (5 μM FXR-LBD) had an EC_50_ value of 60 ± 4 μM. (All experiments were repeated independently four times in duplicates and yielded uniform results; Fig. 7A,B represent one repeat).

In the thermal shift experiments, we used 5 μM of recombinant FXR-LBD protein (residues 244–472) and titrated the FXR agonist GW4064 from 1 μM to 500 μM to find the optimal concentration for competitive measurement. The melting curve revealed a robust stabilization of the FXR-LBD protein starting from 100 μM GW4064 which corresponds to a ratio of 20:1 between ligand and protein (Fig. 8). In absence of protein, GW4064 produced no effect which confirmed that GW4064 does not interact with SYPRO Orange and that the method is suitable to detect a stabilizing effect on the FXR-LBD (Fig. 8).

**Figure 9:**
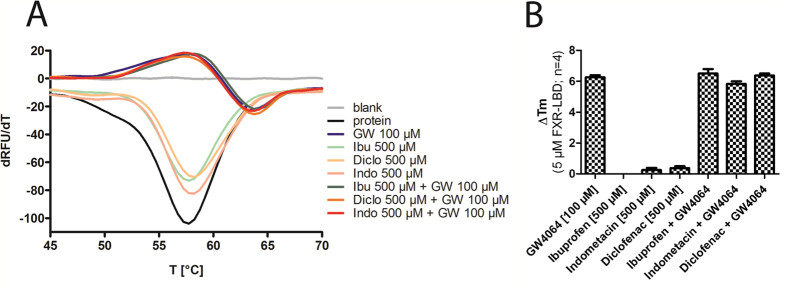
Thermal shift experiments with NSAIDs. (**A**) Ibuprofen, diclofenac, and indometacin (all 500 μM, ratio to protein 100:1) alone did not stabilize the FXR-LBD and did not antagonize the stabilization of the FXR-LBD by GW4064 (100 μM, ratio 20:1) when assessed in competition. (**B**) GW4064 produced a thermal shift of 6 °C. NSAIDs had no effect alone and did not affect the thermal shift caused by GW4064 when tested in competition. (All experiments were repeated independently four times in duplicates and yielded uniform results; Fig. 9A represents one repeat).

Next, we determined the stability of the FXR-LBD in presence of the NSAIDs ibuprofen, diclofenac or indometacin at high concentrations (500 μM each, 100:1 ratio compound to protein). The melting curve showed no significant difference between FXR-LBD protein alone and protein with NSAID (Fig. 9). Finally, we investigated a potential competitive activity of the NSAIDs on the GW4064 dependent stabilization of the FXR-LBD by co-incubation of GW4064 with ibuprofen, diclofenac, or indometacin. To observe even a slight antagonistic effect we used the lowest concentration of GW4064 (100 μM) that had stabilized the protein and high concentrations of the NSAIDs (500 μM). Again, no difference was observed in the melting curve of the FXR-LBD in presence of GW4064 or GW4064 and NSAIDs (Fig. 9). All thermal shift experiments were repeated independently four times and yielded uniform results (Supporting Information).

The thermal shift experiments revealed no effects of the NSAIDs on the FXR-LBD which confirmed our *in vitro* data and our assumption that NSAIDs do not interact with the FXR-LBD and do not antagonize FXR activation by agonists.

## Discussion

Our results are in strong contrast to the reported FXR antagonistic activity of NSAIDs that was observed by Lu *et al*. and in our opinion the data published by Lu *et al*. has several limitations.

First, the use of CDCA as positive control FXR agonist in a reporter gene assay is unfavorable since CDCA is only moderately potent as FXR agonist and at the same time exhibits considerable toxicity. Therefore it is very difficult to generate constant and reproducible FXR activation with CDCA. Since the EC_50_ value of CDCA in reporter gene assays is around 10 μM high concentrations of CDCA are required for a robust FXR activation but such high concentrations also affect the viability of the assay cells.

The high toxicity of CDCA is further enhanced when it is used as competitive agent for antagonistic testing. In a reporter gene assay, antagonistic activity can be determined as a reduced activity of the reporter gene but the control gene—provided a control gene is included—should not be affected by the antagonistic agent since this would indicate a toxic effect rather than antagonism. Hence, it is thinkable that what Lu *et al*. interpreted as antagonism was merely toxicity. In congruence with this assumption, we observed very similar values for simple cell viability when we treated the same cell line with the same compounds as Lu *et al*. have reported as antagonistic activity in their reporter gene (Fig. 5). Eventually, this toxic effect was not taken into consideration.

As a further limitation, Lu *et al*. did not report how many repeats and replicates of their experiments have been conducted and a statistical analysis is missing. With the high variations that must be expected with CDCA (50 μM) as positive control it is also possible that fluctuations contributed to the results.

In our test systems that have intensively been validated and yield robust and reproducible results, no activity was present for the NSAIDs ibuprofen, indometacin, and diclofenac, neither alone nor in competition with the well-known FXR agonists GW4064, OCA or CDCA at typical concentrations. Even at quite low concentrations of CDCA (20 μM) as competitor that should benefit a possible antagonistic activity, no effect was detectable. However, we observed considerable toxicity for diclofenac (30 μM) and indometacin (30 μM) which might easily be misinterpreted as antagonism in case no control gene is used for normalization.

For characterization of nuclear receptor ligands, quantification of target gene expression in ‘native cells’, i.e. not transfected cells is very important. However, given the highly complex network of nuclear receptor signaling, the selection of suitable target genes is essential to obtain results that qualify for a sound interpretation. Therefore, direct target genes should be selected that are affected by the nuclear receptor in first instance and not via a secondary pathway after the expression of a direct target gene has changed. In addition, target genes are required that are only or at least predominantly affected by the nuclear receptor in question.

Many direct target genes of FXR have been reported including the small hetero-dimer partner (SHP) and several bile acid transporters such as the bile salt export protein (BSEP). However, the regulation of the unusual nuclear receptor SHP is very complex and involves many other nuclear receptors that can affect the expression of SHP. The promoter region of the SHP gene contains response elements for e.g. the nuclear receptors hepatocyte nuclear factor 4α (HNF4α), liver receptor-homolog 1 (LRH-1), estrogen receptor α (ERα), liver X receptor α (LXRα), pregnane X receptor (PXR), peroxisome proliferator-activated receptor γ (PPARγ) and FXR^20^,^21^. It has to be noted that these different nuclear receptors can also compete at the promoter region and affect each other which makes the interpretation even more complex. Although it is naturally very important to know what effect a FXR ligand exerts on SHP expression, an effect on SHP expression cannot automatically be interpreted as activity on FXR. The same holds true for secondary target genes of FXR such as cholesterol 7α-hydroxylase (CYP7A1) that have very important physiological effects but are regulated by many different signaling pathways including several nuclear receptors. CYP7A1 is regulated e.g. by nuclear receptors LXR, HNF4α, and SHP and therefore transcriptional effects on CYP7A1 can result from activation or repression of various molecular targets^21^. A considerably more specific target gene is BSEP which is almost exclusively regulated by FXR^21^,^22^. Therefore, effects on the expression of BSEP offer more insights into the activity of an agent on FXR.

Lu *et al*. have quantified the effect of the NSAIDs ibuprofen and indometacin on the expression of SHP and CYP7A1 induced by 50 μM CDCA in HepG2 cells. They report an antagonistic activity of the NSAIDs on both genes. In case of SHP this results in reduced mRNA levels compared to CDCA alone and in case of CYP7A1 leads to enhanced expression since CYP7A1 is not directly affected by FXR but dependent from SHP activity. However, it is confounding that ibuprofen and indometacin have significantly different efficacy in these experiments although they were equally potent in all other *in vitro* assays reported by Lu *et al*.^12^. The fact that indometacin was reported to induce the expression of CYP7A1 by a factor 9 while ibuprofen only produced a 1.7-fold increase in mRNA levels indicates that other pathways are involved. Based on the complex regulation of SHP and CYP7A1 and the heterogeneous data the effects on SHP and CYP7A1 expression reported by Lu *et al*. are difficult to interpret.

To obtain more specific data for transcriptional effects via FXR we selected BSEP as target gene for our experiments. In addition, we evaluated a possible effect on SHP although such effect might be explained by activity on various targets. However, we did not observe any antagonistic effect on BSEP or SHP expression by ibuprofen, diclofenac or indometacin in competition with CDCA which strongly confirmed our data from the reporter gene assays.

Hence, our *in vitro* data clearly indicates that the NSAIDs ibuprofen, indometacin, and diclofenac have no functional activity on FXR. In addition, thermal shift experiments showed that the NSAIDs do not bind to the purified FXR-LBD under cell-free conditions. We therefore conclude that reduced FXR activation by CDCA in presence of high concentrations of some NSAIDs (especially diclofenac) is not a cause but rather a consequence of toxicity and that NSAIDs do not interact with farnesoid X receptor.

## Materials and Methods

### Test compounds

NSAIDs ibuprofen (CAS: 15687-27-1, Alfa Aesar, Karlsruhe, Germany), indometacin (CAS: 53-86-1, Alfa Aesar) and diclofenac sodium (CAS: 15307-79-6, Acros Organics, Schwerte, Germany) as well as FXR agonists GW4064 (CAS: 278779-30-9, Sigma Aldrich, St. Louis, MO, USA), OCA (CAS: 459789-99-2, Biomol, Hamburg, Germany) and CDCA (CAS: 474-25-9, Sigma Aldrich) were purchased. Identity of the compounds was confirmed by ^1^H and ^13^C NMR. All compounds had a purity >98% according to manufacturer and were used for *in vitro* test systems without further purification.

### Hybrid reporter gene assay (Gal4-FXR)

COS-7 cells were grown in DMEM high glucose, supplemented with 10% FCS, sodium pyruvate (1 mM), penicillin (100 U/mL) and streptomycin (100 μg/mL) at 37 °C and 5% CO_2_.

The Gal4-fusion receptor plasmid pFA-CMV-hFXR-LBD containing the hinge region and ligand binding domain (LBD) of FXR was constructed by integrating cDNA fragments obtained from PCR amplification of human monocytes into the SmaI/XbaI cleavage site of the pFA-CMV vector (Stratagene, La Jolla, CA, USA) and has already been published^23^. The cDNA fragment consists of bps 565–1416. Frame and sequence of the fusion receptor was verified by sequencing. pFR-Luc (Stratagene) was used as reporter plasmid and pRL-SV40 (Promega) for normalization of transfection efficiency and cell growth.

The day before transfection, COS-7 cells were seeded in 96-well plates (3 · 10^4^ cells/well). Transient transfection was carried out using Lipofectamine LTX reagent (Invitrogen, Carlsbad, CA, USA) according to the manufacturer’s protocol with pFR-Luc (Stratagene), pRL-SV40 (Promega) and pFA-CMV-hFXR-LBD. 5 h after transfection, medium was changed to DMEM without phenol red, supplemented with sodium pyruvate (1 mM), penicillin (100 U/mL), streptomycin (100 μg/mL) L-glutamine (2 mM), now additionally containing 0.1% DMSO and the respective test compound or 0.1% DMSO alone as untreated control. Each concentration was tested in triplicates and each experiment was repeated independently at least four times. Following overnight (12–14 h) incubation with the test compounds, cells were assayed for luciferase activity using Dual-GloTM Luciferase Assay System (Promega) according to the manufacturer’s protocol. Luminescence was measured with an Infinite M200 luminometer (Tecan Deutschland GmbH). Normalization of transfection efficacy and cell growth was done by division of firefly luciferase data by renilla luciferase data and multiplying the value by 1000 resulting in relative light units (RLU). Fold activation was obtained by dividing the mean RLU of a test compound at a respective concentration by the mean RLU of untreated control. Relative activation was obtained by dividing the fold activation of a test compound at a respective concentration by the fold activation of FXR full agonist GW4064 at 3 μM.

The assay was validated with FXR agonists GW4064 (EC_50_ = 0.22 ± 0.04 μM, 3 μM defined as 100%), OCA (EC_50_ = 0.54 ± 0.05 μM, 110 ± 3% max.) and CDCA (EC_50_ = 14.5 ± 0.8 μM, 111 ± 5% max.).

### Full-length FXR reporter gene assay (flFXR)

HeLa cells were grown in DMEM high glucose supplemented with 10% FCS, SP (1 mM), penicillin (100 U/mL) and streptomycin (100 μg/mL) at 37 °C and 5% CO_2_.

pcDNA3-hFXR contains the sequence of human FXR and was already published elsewhere^24^, pGL3basic (Promega Corporation, Fitchburg, WI, USA) was used as a reporter plasmid, with a shortened construct of the promotor of the bile salt export protein (BSEP, sequence of construct from^25^) cloned into the SacI/NheI cleavage site in front of the luciferase gene. pRL-SV40 (Promega Corporation) was transfected as a control for normalization of transfection efficiency and cell growth. pSG5-hRXR was already published elsewhere^26^ as well.

24 h before transfection, HeLa cells were seeded in 96-well plates (8 · 10^3^ cells/well). 3,5 h before transfection, medium was changed to DMEM high glucose, supplemented with sodium pyruvate (1 mM), penicillin (100 U/mL), streptomycin (100 μg/mL) and 0.5% charcoal-stripped FCS. Transient transfection of HeLa cells with BSEP-pGL3, pRL-SV40 and the expression plasmids pcDNA3-hFXR and pSG5-hRXR was carried out using the calcium phosphate method. 16 h after transfection, medium was changed to DMEM high glucose, supplemented with sodium pyruvate (1 mM), penicillin (100 U/mL), streptomycin (100 μg/mL) and 0.5% charcoal-stripped FCS. 24 h after transfection, medium was changed to DMEM without phenol red, supplemented with sodium pyruvate (1 mM), penicillin (100 U/mL), streptomycin (100 μg/mL), L-glutamine (2 mM) and 0.5% charcoal-stripped FCS, now additionally containing 0.1% DMSO and the respective test compound or 0.1% DMSO alone as untreated control. Each concentration was tested in triplicates and each experiment was repeated independently at least five times. Following 24 h incubation with the test compounds, cells were assayed for luciferase activity using Dual-GloTM Luciferase Assay System (Promega Corporation) according to the manufacturer’s protocol. Luminescence was measured with a Tecan Infinite M200 luminometer (Tecan Deutschland GmbH, Crailsheim, Germany). Normalization of transfection efficiency and cell growth was done by division of firefly luciferase data by renilla luciferase data and multiplying the value by 1000 resulting in relative light units (RLU). Fold activation was obtained by dividing the mean RLU of the tested compound at a respective concentration by the mean RLU of untreated control. Relative activation was obtained by dividing the fold activation of the tested compound at a respective concentration by the fold activation of FXR full agonist GW4064 at 3 μM.

The assay was validated with FXR agonists GW4064 (EC_50_ = 0.51 ± 0.16 μM, 3 μM defined as 100%), OCA (EC_50_ = 0.16 ± 0.02 μM, 87 ± 3% max.) and CDCA (EC_50_ = 18 ± 1 μM, 88 ± 3% max.).

### Cell proliferation assay (WST-1)

WST-1 assay (Roche Diagnostics International AG, Rotkreuz, Switzerland) was performed according to manufacturer’s protocol. In brief, HepG2 cells were seeded in DMEM high glucose, supplemented with sodium pyruvate (1 mM), penicillin (100 U/mL), streptomycin (100 μg/mL) and 10% FCS in 96-well plates (3 · 10^4^ cells/well). HEK293T cells were seeded in DMEM high glucose, supplemented with sodium pyruvate (1 mM), penicillin (100 U/mL), streptomycin (100 μg/mL) and 10% FCS in 96-well plates (2,5 · 10^4^ cells/well). After 24 h, medium was changed to DMEM high glucose, supplemented with penicillin (100 U/mL), streptomycin (100 μg/mL) and 1% charcoal stripped FCS and cells were incubated with varying concentrations of test compounds, Revlotron (100 μM) as positive control and DMEM/0,1% DMSO as negative control. After 48 h, WST reagent (Roche Diagnostics International AG) was added to each well according to manufacturer’s instructions. For determination of combined toxicity of NSAIDs and CDCA in HEK cells WST reagent was added after 24 h and to determine short-term toxicity, viability of HepG2 cells was measured after 6 h incubation. After 45 min incubation, absorption (450 nm/reference: 620 nm) was determined with a Tecan Infinite M200 luminometer (Tecan Deutschland GmbH). Each experiment was repeated at least four times in triplicates.

### FXR target gene quantification by qRT-PCR

HepG2 cells were seeded in DMEM high glucose, supplemented with 10% FCS, SP (1 mM), penicillin (100 U/mL) and streptomycin (100 μg/mL) at 37 °C and 5% CO_2_ in 6-well plates (2 · 10^6^ per well). 24 h after seeding, medium was changed to MEM, supplemented with 1% charcoal-stripped FCS, penicillin (100 U/mL), streptomycin (100 μg/mL) and L-glutamine (2 mM). After additional 24 h, medium was changed again to MEM, now additionally containing 0.1% DMSO and the respective test compounds or 0.1% DMSO alone as untreated control. Cells were incubated with the test compounds for 6 h, harvested, washed with cold phosphate buffered saline (PBS) and then directly used for RNA extraction.

Total RNA was extracted from HepG2 cells by the Total RNA Mini Kit (R6834-02, Omega Bio-Tek, Inc., Norcross, GA, USA). RNA was reverse-transcribed into cDNA using the High-Capacity cDNA Reverse Transcription Kit (4368814, Thermo Fischer Scientific, Inc., Waltham, MA, USA) with 2 μg RNA according to the manufacturer’s protocol.

FXR target gene expression was evaluated by quantitative PCR analysis with a StepOnePlusTM System (Life Technologies) using PowerSYBRGreen (Life Technologies; 12.5 μL per well) and the following primers^17^ (300 nM each): SHP: 5′-GCTGTCTGGAGTCCTTCTGG (forward) and 5′-CCAATGATAGGGCGAAAGAAGAG (reverse); BSEP: 5′-CATGGTGCAAGAAGTGCTGAGT (forward) and 5′-AAGCGATGAGCAACTGAAATGAT (reverse). Results were normalized to GAPDH Ct values. Sequences of the GAPDH primers were as follows: 5′-ATATGATTCCACCCATGGCA (forward) and 5′- GATGATGACCCTTTTGGCTC (reverse). Each sample was set up in duplicates and repeated in at least four independent experiments. The expression was quantified by the comparative ΔΔCt method.

### Thermal shift

Thermal Shift assay was performed in clear 96-well plates (Invitrogen) using SYPRO Orange (Invitrogen Darmstadt, Germany) as dye. 10 μL of test compound (GW4064: final concentration 1 μM–500 μM, 100 μM for competitive testing; NSAIDs: 500 μM final concentration) in assay buffer (10 mM TRIS (pH 8.3), 5 mM DTT, 0.5 mM EDTA, 100 mM NaCl) were mixed with 10 μL of protein (final protein concentration 5 μM) in assay buffer and 5 μL of SYPRO Orange (5 × final concentration) in assay buffer. Temperature-dependent fluorescence increase reporting protein denaturation was measured in duplicates in an ICycler (Bio-Rad) from 20 to 90 °C in steps of 0.2 °C per minute at 300 nm excitation and 570 nm emission wavelength. Each experiment was independently repeated four times. The first derivative of the melting curves was calculated using the Graph Pad Prism 5 software.

### Western blotting

For the Western Blot analysis, HeLa cells were prepared, transfected and incubated as described for the full-length FXR reporter gene assay. Cells were then harvested with TE-buffer (10 mM Tris-Cl, pH 7.5, 1 mM EDTA), collected and centrifuged (3000 rpm for 5 minutes). The supernatants were discarded, the pellets were washed in PBS buffer (1.4 mM NaCl, 2.7 mM KCl, 1.8 mM KH_2_PO_4_, 10 mM Na_2_HPO_4_, pH 7.4) once, re-suspended in 18 μL PBS Buffer and mixed with 6 μL Laemmli buffer (120 mM Tris-Cl, pH 6.8, 20% glycerol, 4% SDS, 0.02% bromophenol blue). The mixtures were denatured by heating to 95 °C for 5 minutes.

For the SDS PAGE, 1 mm Gels (NuPAGE 4–12%) with 15 wells from Invitrogen were used. 10 μL of each sample were loaded on the gel and 3.5 μL PageRuler NIR prestained (Thermo Scientific, Waltham, MA, USA) was used. As running buffer, MES buffer (50 mM MES, 50 mM Tris base, 1 mM EDTA, 0.1% (w/v) SDS) was used.

For the Western Blot, the XCell II™ Blot Module CE Mark (Life Technologies, Darmstadt, Germany) was used. A PVDF membrane (Merck Millipore, Darmstadt, Germany) was activated with 100% Methanol (5 seconds), washed with ultrapure water and soaked in WET blot buffer (195 mM glycine, 240 mM Tris, pH 9.2). After blotting for one hour, the membrane was blocked with 0.2% I-Block™ reagent in PBS buffer with 0.1% Tween20® for one hour at room temperature and then incubated for one hour with the primary antibody-0.2% I-Block™ PBS buffer (A5441 from Sigma Aldrich) at 4 °C. The membrane was washed three times for 10 minutes with 0.2% I-Block™ PBS buffer before it was incubated for one hour with the second antibody-0.2% I-Block™ PBS buffer (donkey-anti-mouse IRDye680LT from Li-Cor, Lincoln, NB, USA). After incubation, the membrane was washed two times for 10 minutes with 0.2% I-Block™ PBS buffer and once with PBS Puffer. The blot was stored at 4 °C in PBS buffer and measured at 700 and 800 nm with an Odyssey CLx (Li-Cor).

## Additional Information

**How to cite this article**: Schmidt, J. *et al*. NSAIDs Ibuprofen, Indometacin, and Diclofenac do not interact with Farnesoid X Receptor. *Sci. Rep*. **5**, 14782; doi: 10.1038/srep14782 (2015).

## Supplementary Material

Supplementary Information

## Figures and Tables

**Figure 1:**
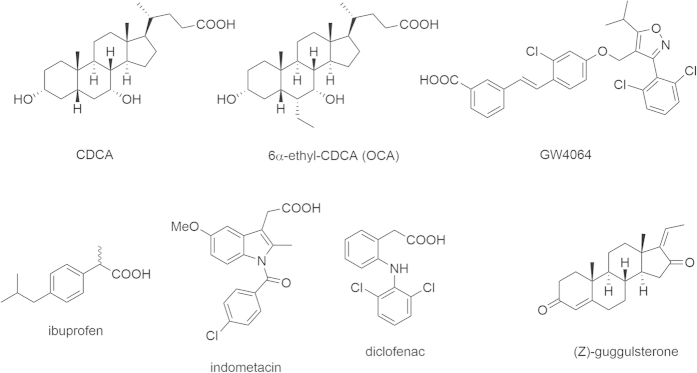
NSAIDs ibuprofen, indometacin and diclofenac, common FXR agonists CDCA, OCA and GW4064 as well as the SBARM (Z)-guggulsterone which is commonly used as reference FXR antagonist.
