# Supplementary material for: NSAIDs Ibuprofen, Indometacin, and Diclofenac do not interact with Farnesoid X Receptor
Source: Sci Rep. 2015 Oct 1;5:14782. doi: 10.1038/srep14782 (PMC4589779; doi:10.1038/srep14782)

NSAIDS IBUPROFEN, INDOMETACIN, AND DICLOFENAC DO NOT INTERACT WITH FARNESOID X RECEPTOR

Jurema Schmidt[1], Franca-Maria Klingler[1], Ewgenji Proschak[1], Dieter Steinhilber[1], Manfred Schubert-Zsilavecz[1], Daniel Merk[1]*

[1] Institute of Pharmaceutical Chemistry, Goethe University Frankfurt, Max-von-Laue-Str. 9, 60438 Frankfurt, Germany

* merk@pharmchem.uni-frankfurt.de

- SUPPORTING INFORMATION -

Thermal shift data (n=4, each figure represents one independent repeat)

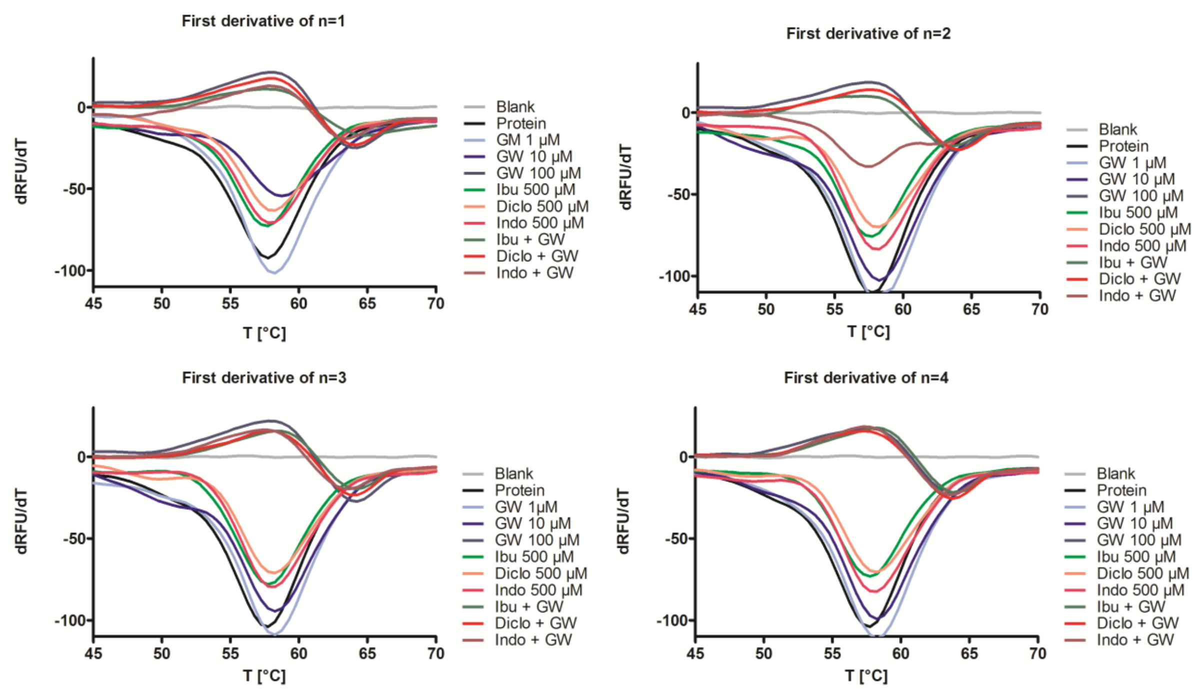

Supplement: Supplementary Information [file srep14782-s1.pdf]
